# Anatomical investigation of potential contacts between climbing fibers and cerebellar Golgi cells in the mouse

**DOI:** 10.3389/fncir.2013.00059

**Published:** 2013-04-08

**Authors:** Elisa Galliano, Marco Baratella, Martina Sgritta, Tom J. H. Ruigrok, Elize D. Haasdijk, Freek E. Hoebeek, Egidio D'Angelo, Dick Jaarsma, Chris I. De Zeeuw

**Affiliations:** ^1^Department of Neuroscience, Erasmus Medical Centre RotterdamRotterdam, Netherlands; ^2^Department of Neuroscience and Brain Connectivity Center, University of Pavia and IRCCS C. MondinoPavia, Italy; ^3^Netherlands Institute for Neuroscience, Royal Netherlands Academy of Arts and SciencesAmsterdam, Netherlands

**Keywords:** cerebellum, climbing fiber, Golgi cells, synapse, confocal microscopy

## Abstract

Climbing fibers (CFs) originating in the inferior olive (IO) constitute one of the main inputs to the cerebellum. In the mammalian cerebellar cortex each of them climbs into the dendritic tree of up to 10 Purkinje cells (PCs) where they make hundreds of synaptic contacts and elicit the so-called all-or-none complex spikes controlling the output. While it has been proven that CFs contact molecular layer interneurons (MLIs) via spillover mechanisms, it remains to be elucidated to what extent CFs contact the main type of interneuron in the granular layer, i.e., the Golgi cells (GoCs). This issue is particularly relevant, because direct contacts would imply that CFs can also control computations at the input stage of the cerebellar cortical network. Here, we performed a systematic morphological investigation of labeled CFs and GoCs at the light microscopic level following their path and localization through the neuropil in both the granular and molecular layer. Whereas in the molecular layer the appositions of CFs to PCs and MLIs were prominent and numerous, those to cell-bodies and dendrites of GoCs in both the granular layer and molecular layer were virtually absent. Our results argue against the functional significance of direct synaptic contacts between CFs and interneurons at the input stage, but support those at the output stage.

## Introduction

Classically, the olivo-cerebellar system is believed to be an online comparator that calculates the difference between a desired and an executed movement via its highly organized and preserved cellular network and forwards the appropriate modification through its projections to the brainstem (Marr, 1969; Albus, 1971). Among the numerous cell types in the cerebellar cortical network, the Purkinje cell (PC) is traditionally thought to be the most important, because it is the only one to directly receive both signals on movement context and signals on sensory feedback (Bloedel and Bracha, 1998; Schmolesky et al., 2002), and because it constitutes the sole output of the cerebellar cortex so as to adjust movements.

Information about the desired and executed behavior reaches the cerebellar cortex via two main types of fibers. These are the so-called climbing fibers (CFs), which all originate from the inferior olivary nucleus (IO), and the mossy fibers (MFs), which can be derived from many other sources in the brainstem (Ramon y Cajal, 1995). The MFs provide inputs on the context of planned and ongoing movements and are connected to the PCs through a di-synaptic pathway via cerebellar granule cells (GCs), which in turn innervate the PCs by their parallel fibers. The CFs on the other hand probably provide the relevant feedback signals for adjusting the amplitude and timing of movements (De Zeeuw et al., 2011; Gao et al., 2012). Single CFs make direct and numerous synaptic contacts with PCs. A classical model of cerebellar functioning postulates that the CFs provide the required error signals encoding the difference between the executed and desired movement and thereby guide motor learning (Marr, 1969; Albus, 1971). More recently, it has been proposed that the CFs do not only evoke their feedback via their direct contacts on the PC dendritic tree, but also through extrasynaptic effects via the molecular layer interneurons (MLIs) (Szapiro and Barbour, 2007; Gao et al., 2012; Mathews et al., 2012). Moreover, in principle it is possible that collaterals of the olivary axons also contact Golgi cells (GoCs), which provide direct inhibition onto the GCs (Galliano et al., 2010). Branches of olivary axons, named Scheibel collaterals (Scheibel and Scheibel, 1954), are known to travel through the densely populated granule cell layer (GL) and thus can approach cell-bodies and/or dendrites of GoCs (Hamori and Szentagothai, 1966a; Palay and Chan-Palay, 1974; Shinoda et al., 2000). Indeed, two electrophysiological studies confirmed an effect of olivary activation on GoC firing, but they were unable to elucidate whether these effects were mono-synaptic or multi-synaptic (Schulman and Bloom, 1981; Xu and Edgley, 2008). Here, we took various morphological approaches to shed light on the question as to what extent CFs may also directly contact GoCs. We injected a fluorescent anterograde tracer in the IO in mutant mice that express eGFP in their glycinergic neurons (GlyT2-eGFP) so as to enable immunofluorescent identification of both CF terminals and GoCs in the same material and subsequently quantify their appositions. For comparison we also labeled the PCs with Calbindin and the MLIs'cell-bodies with DAPI in combination with VGlut2 staining of CFs terminals, allowing us to compare the density of appositions of CFs onto GoCs with those onto PCs and MLIs.

## Methods

### Animals

We used mice of both genders older than 20 days, either inbred C57BL/6 mice provided by Harlan Laboratories (The Netherlands) or transgenic mice that specifically express enhanced green fluorescent protein under the control of the glycine transporter type 2 promoter (GlyT2-EGFP). The GlyT2-EGFP were generated and kindly provided by Dr. Fritschy (Zeilhofer et al., 2005), and all experimental animals were bred at the Erasmus MC breeding facility by backcrossing with C57BL/6. All experiments were performed in accordance with the guidelines for animal experiments of the respective universities and the Dutch national legislation.

### Olivary injections

Injections of the neuro-anatomical tracer biotin dextran amine (BDA) in the IO were performed as previously described in rats (Pijpers et al., 2005). Briefly, mice were anaesthetized (~1.5% isofluorane in O_2_, buprenorphine 0.05 mg/kg subcutaneous, and rymadyl 5 mg/kg, subcutaneous) and placed in a stereotactic head holder. The dorsal side of the head and neck was exposed using a midline incision of the skin (running from lambda to processus spinosus of C2). Neck muscles were split longitudinally and laterally with a spreader. The atlanto-occipital membrane and dura mater were opened to gain a direct view of the caudal part of the medulla oblongata and caudal part of lobule IX of the cerebellum. From this position the IO could be well approached by a micromanipulator driven glass micropipette (tip diameter: 5–10 μm) filled with the neuro-anatomical tracer BDA (10,000 mW) dissolved in 0.5 μl 2% NaCl using stereotactic coordinates. Location of injection was determined by use of a stereotactic mouse atlas (Paxinos and Franklin, 2004) and typical electrophysiological characteristics of the IO neurons (i.e., extracellular action potential consisting of a usually negative-positive going spike that is followed by a negative wave lasting about 5 msec or by several small spikelets and an overall spike frequency of ~1 Hz; see example trace in Figure 2A and Ruigrok et al., 1995). Afterwards, the dura was placed back, the left, and right muscles were attached to each other in layers and the skin was sutured. After 5 days of recovery in their home cage mice were perfused and processed for immunohistochemistry.

### Immunofluorescence

The animals were deeply anesthetized by intraperitoneal administration sodium pentobarbital (Nembutal) and perfused through the ascending aorta with saline followed by 4% paraformaldehyde in 0.12 M phosphate buffer (PB). Brains were removed, immersed in the same fixative for 1.5 h at room temperature, and subsequently cryoprotected in 10% sucrose in PB solution and embedded in a gelatin block (12% in PB). Blocks were postfixed in 10% paraformaldehyde and 30% sucrose solution for 1.5 h at room temperature; subsequently they were immersed in 30% sucrose overnight at 4°C. Coronal or sagittal sections were cut at 40 μm with a freezing microtome (Leica SM 2000 R), and then collected in PBS. Sections were rinsed in 0.1 M PB and incubated for 2 h in 10 mM Na-citrate at 80°C. After the antigen retrieval the sections were washed with TBS. Free-floating sections were blocked against non-specific antibody binding with a pre-incubation step of 1 h at room temperature, in a TBS buffer containing 10% normal horse serum and 0.5% Triton X-100. Free-floating section were then incubated for 48–72 h at 4°C in a mixture of primary antibodies diluted in TBS buffer containing 2% normal horse serum and 0.4% Triton. Sections were washed and incubated for 1.5–2 h at room temperature in a mixture of secondary antibodies (10 ml buffer: 200 μl AB) coupled to a fluorochrome. Sections were washed again, mounted onto gelatin-coated slides, air-dried, and cover-slipped with a mounting medium for Fluorescence (Vectrashield H-1000). Primary antibodies used were mouse anti-mGluR2 (1:1000, AbCam), Guinea pig anti-VGlut2 (1:1000, Millipore), mouse anti-Calbindin (1:7000, Sigma). We used FITC, Alexa Fluor488, Cy3, and Cy5 conjugated anti-mouse IgG as secondary antibodies (Jackson ImmunoResearch) (Hossaini et al., 2011). To label BDA we used alexa Fluor568 Streptavidin. In case of section from GFP mouse and tracing VGLUT was labeled with Cy5. Generally sections were counterstained with DAPI (1:100,000, Invitrogen).

### Selection of appropriate markers for GoCs and CFs

To study the connectivity between GoCs and CFs we selected two reliable GoC markers: the metabotropic glutamate receptor type 2 (mGluR2), which stains 83.5% of the entire GoC population (Neki et al., 1996; Simat et al., 2007); and the glycine transporter type 2 (GlyT2), which is expressed by 94.5% of GoCs (Simat et al., 2007) and has been coupled with EGFP in a transgenic animal [GlyT2-EGFP animals, (Zeilhofer et al., 2005)]. Together these markers encompass the complete population of GoCs (Simat et al., 2007). CF synaptic terminals in the molecular layer were identified using vesicular glutamate transporter type 2 (VGluT2) immunohistochemistry (Kaneko et al., 2002). In the granular layer, however, VGluT2 immunostaining is not exclusive for CFs, since a subset of MFs also colocalize with VGluT2 staining (Kaneko et al., 2002). To unequivocally identify CFs in the granular layer we injected the IO with the anterograde tracer BDA and, in order to unequivocally prove the presence of a synapse, also stained this tissue for VGluT2. Finally, we used Calbindin staining to identify PCs and DAPI staining to identify cell-bodies of MLIs in the molecular layer.

### Data analysis

Images (512 × 512 or 1024 × 1024 pixels) were obtained using the confocal laser scanning microscope Zeiss LSM 700 (Zeiss, Jena, Germany) equipped with 10×, 20×, 40×, 63× lenses. All the samples were analyzed making z-stacks with an interval of 0.3–0.45 μm and the presence of CF-GO contact was investigated analyzing each z-plane.

The quantification in 10 μm thick sections was performed in 50 × 50 × 10 mm portions of ML and GL (*n* = 30, *N* = 3 for both layers). Each maximum intensity projection image (aligned configuration) was then processed by rotating 90° clockwise (rotated configuration) or translating 25 pixels to the right (translated configuration) either the VgluT2 channel (ML) or the GlyT2 channel (GL). The resulting 180 images (90 ML and 90 GL) were shuffled, renamed and blindly analyzed in order to quantify the number of colocalizations (i.e., spatial overlap of two or three colors) between VGluT2 + GlyT2/mGluR2 (ML), VGlut2 + BDA + GlyT2, and BDA + GlyT2 (GL). Differences between the three configurations (aligned, rotated, and translated; see scheme in Figure 4) were statistically analyzed with a One-Way ANOVA with Tukey *post-hoc* corrections.

Antibody dilutions and settings of the confocal microscopy (CM) were optimized to avoid bleed-through of one fluorophore into the other. Fluorophores with overlapping excitation/emission spectra (e.g., Alexa488 and Cy3) were collected in different tracks. Routinely, confocal stacks were collected in dual tract mode with DAPI and orange/red fluorophore (Cy3, Alexa555, Alexa 568) in one tract (wave lengths 405 and 555, filter settings: *LP* = 560, *SP* = 490) and green (FITC, Alexa) and infra red in the other tract (wave lengths 488 and 639, filter settings: *LP* = 640, *SP* = 555). To avoid false negatives only established markers that produce strong signal were selected. To avoid chromatic aberration the confocal microscope was routinely calibrated for differences in focal lengths.

Offline analysis of both thin and thick sections was performed with LSM Image browser and Image J software packages.

## Results

### Absence of co-localization of mGluR2 and VGluT2 in the ML

**Figure 1:**
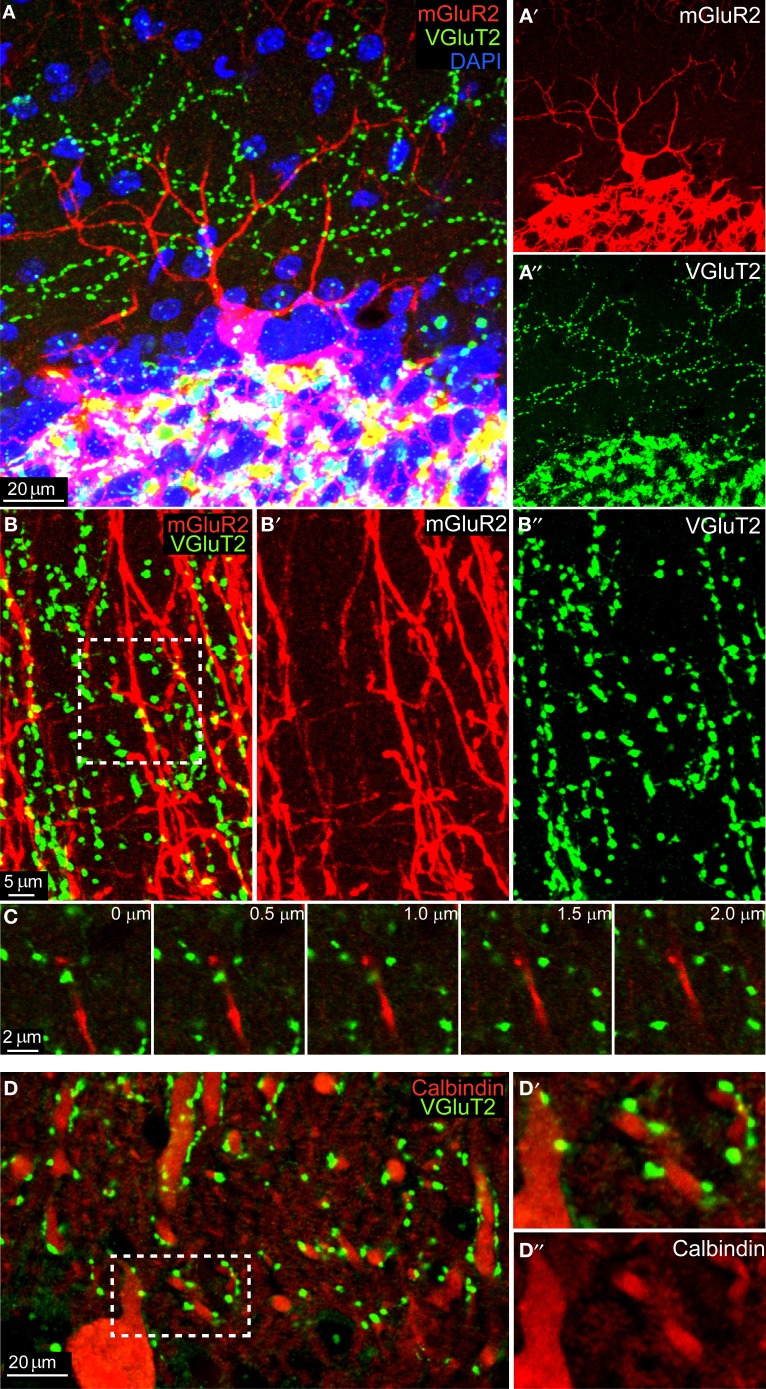
**No colocalization between mGluR2-marked GoC and VGlut2 in the ML. (A)** Maximum intensity projection of a 20 μm-thick sagittal section of an mGlur2-stained GoC (red) located in the upper GL (identifiable through the extremely dense DAPI staining, in blue). Its dendrite ascends to the ML, where punctuate staining indicates VGlut2. The red and green channels are split and presented separately in, respectively, **(A')** and **(A”)**. Note the mismatch of the two signals. **(B)** High-magnification of the ML in coronal section (maximum intensity projection of a 2 μm slices), with red GoC dendrites (mGluR2 positive; **B'**) and VGlut2-marked CF endings (green; **B”**). Potential colocalizations are scarce, and when present appear like yellow spots (see arrow). **(C)** Montage in the z-plane with 0.5 μm interval between photographs of the inset indicated in **(B)**. Note how the GoC dendrite (red) and the VGluT2 varicosity (green) lay on different planes, separated by at least 1 μm. **(D)** Maximum intensity projection of a 4.5 μm-thick sagittal section of Calbindin-stained PCs dendrites (red, see detail in **D”**) which abundantly colocalized with VGlut2 (green; **D'**).

We performed mGluR2 and VGluT2 immunohistochemistry in 3 C57BL/6 adult animals and we analyzed both sagittal and coronal sections. As shown in Figure 1A, the mGluR2 antibody reliably stained GoCs (in red, see also Figure 1A′) allowing the visualization of the entire dendritic tree. Similarly, VGlut2 staining (in green, see also Figure 1A″) revealed a typical CF pattern in the ML and MF rosettes in the GL. High-magnification images of the ML showed a clear mis-match between GoCs dendrites and VGluT2 expression (Figures 1B–B″). Occasionally, maximum intensity projections of three-dimensional areas hinted toward a possible co-localization (see inset in Figure 1B), but a careful analysis in the z-plane consistently confirmed the supposed co-localization to be a bi-dimensional artifact, because the two colors were never present at the same depth (see montage in Figure 1C). In contrast, when we analyzed the number of VGluT2 stained terminals on top of Calbindin stained PCs' dendrites we found consistent and abundant co-localization (Figures 1D–D″). Moreover, when we quantified the number of VGluT2 stained terminals on top of DAPI stained cell-bodies of MLI's (*n* = 550) (for examples, see e.g., Figure 1A), we found that 61.3% showed a co-localization using the same criteria. In summary, these data did not provide any evidence of synaptic contacts in the ML layer between mGluR2-positive GoCs and VGlut2-containing CFs, whereas they provided robust evidence for appositions of CFs onto PCs and MLIs.

### Anterograde tracing of CFs also failed to show contacts onto glycinergic GoCs

**Figure 2:**
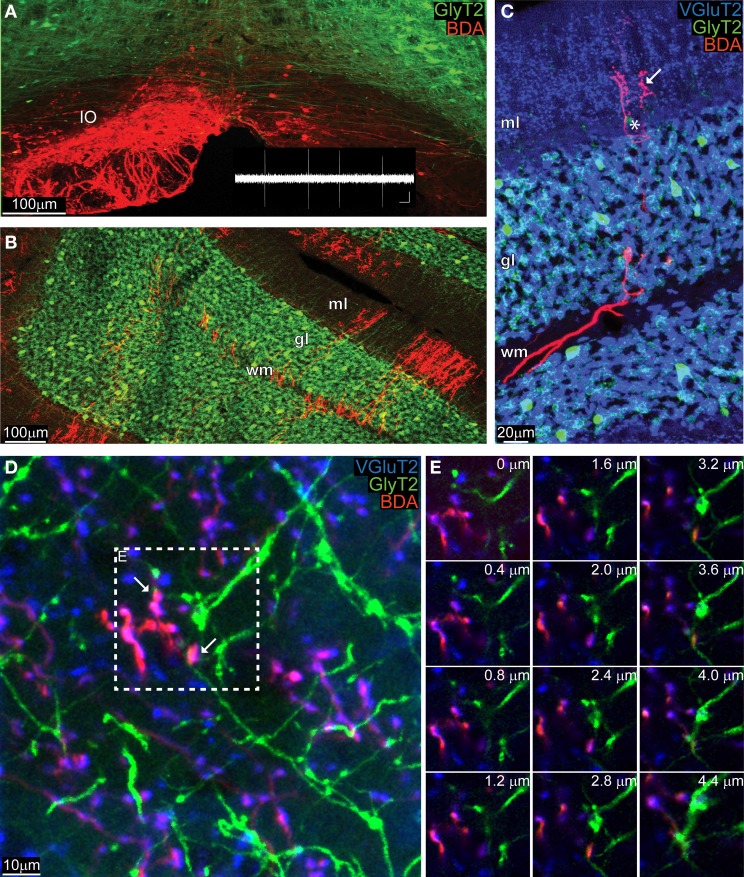
**Anatomically marked CFs co-stain with VGlut2 in the ML, but not with GoC dendrites. (A)** Maximum intensity projection of a low magnification image (10 μm thick) of the IO of a GlyT2-EGFP mouse, in which all glycinergic neurons (GoC included) are green. The animal was unilaterally injected with BDA in the left IO, and the BDA was visualized with a red fluorochrome. The inset in white reproduces a typical olivary neuron firing pattern (scalebar: 0.5 mV, 500 ms). **(B)** View of the cerebellar cortex of the same GlyT2-EGFP mouse presented in **(A)** (maximum intensity projection of a 10 μm thick slice). The BDA along the olivary axon clearly stained CFs, which entered in the white matter (WM), passed through the GL and reached the ML. **(C)** Example picture (maximum intensity projection, 5 μm thick) gathered from a BDA-injected (CFs in red red) GlyT2-EGFP mouse (GoCs in green) additionally stained for the synaptic marker VGluT2 (blue). The CF in the WM and GL remained bright red, while in the ML it co-localized with VGluT2 and circled around a PC (star) and assumed a violet color (arrow). **(D)** Maximum intensity projection at high magnification of the ML (4.4 μm thick), with abundant double labeling BDA-VGlut2 (red and blue), but not with GlyT2 (green). Two potential triple-labelings are indicated by the arrows. **(E)** Montage in the z-plane of the inset presented in **(D)** containing the potential synapses with 0.4 μm interval between photographs. The red and blue channel appear at the same depth, but not the green one, indicating that the GoC dendrite resides in a different plane than the CF terminal.

Five GlyT2-EGFP adult mice received a BDA injection into their IO (Figure 2A, BDA in red), which diffused in the olivary axons all the way up to the cerebellar cortex (Figure 2B) where the typical “climbing” character of CFs was clearly identifiable against the “virtual” dendritic trees of PCs (Figure 2C). Whereas CFs were stained violet in the ML, which is due to the co-localization of BDA (red) with VGluT2 (blue), in the GL CFs remained bright red (Figure 2C). A systematic analysis of such material revealed that any potential triple-localization of the CF (BDA, red), VGluT2 (blue), and GoC dendrites (green) seen in maximum projections (inset in Figure 2D) disappeared when the individual images at various depths were studied (Figure 2E). In total we traced over 200 CFs and analyzed 800 potential BDA and VGluT2 colocalizations with GlyT2. However, a careful analysis in the z-plane revealed that no BDA-VGluT2 staining occurred at the level of a glycinergic GoC dendrite. Taken together with the results described above, our data argue against a CF-GoC connection in the ML.

### CF-GoC synapses also appear absent in the GL

**Figure 3:**
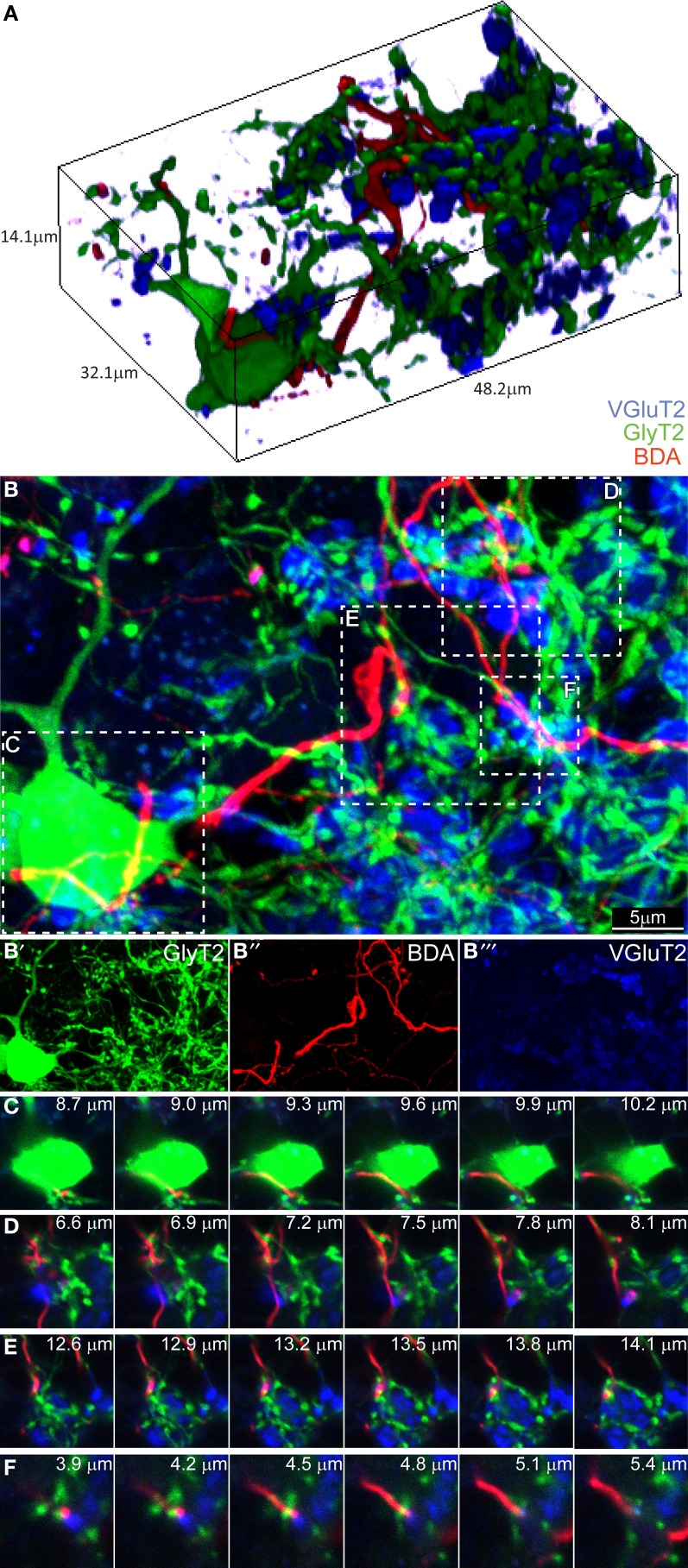
**Three-dimensional analysis of the upper GL fails to discover CF-GoC synapses. (A)** 3D reconstruction of a portion of the upper GL of a BDA-injected (CF, red) GlyT2-EGFP mouse (GoC, green) co-stained for VGluT2, and containing on GoC soma and numerous dendrites, together with one clearly identifiable CF and the typical MF glomerular rosettes (VGlut2 positive, in blue). **(B)** Maximum intensity projection of the 3D image (14.1 μm thick), with below the three individual channels **(B'–B”')**. Five possible triplets are indicated in the insets **(C–F)**. Montages of the five potential synapses analyzed in the z-plane. In all five instances the CF does not co-localize with VGlut2, arguing against the existence of synaptic contacts.

Having found no evidence for a connection in the ML, we proceeded to analyze the GL. We divided it in three subzones (upper third, just below the PCs layer, middle third and lower third). We began focusing on what we believed being the hot-spot for possible CF-GoC contacts due to the presence of the CF “Scheibel” collaterals, i.e., the upper third of the GL (Figure 3). As previously reported for the ML, in the tissue collected from the BDA-injected GlyT2-EGFP mice stained for VGlut2 the potential colocalization of the BDA-marked CFs with VgluT2 and GlyT2 were extremely scarce. Twenty-eight locations appeared promising, but again a thin-section analysis showed that none of these locations had triple labeling with GlyT2, excluding synaptic contacts between CFs and GoCs in this upper GL.

Proceeding deeper toward the white matter, we found 7 combined BDA-VGlut2 positive spots in the middle third of the GL and only 1 in the lower third of the GL, but again none co-stained with GlyT2. In addition, we studied 23 GoC somata and their potential co-localization with BDA-stained CFs, but unlike what we observed in the combined CF-PC and CF-MLI stainings described above, we never found VGluT2-expression in these labeled fibers apposed to GoCs. Figure 3 shows a 3D-representation of the upper GL (Figure 3A) and its maximum intensity projection (Figure 3B; BDA in red, VGluT2 in blue, and glycinergic GoC in green). Four possible contact points, either on the GoC soma (inset C) or at the level of GoC dendrites (insets D–F), were analyzed in detail in the z-plane (Figures 3C–F). Yet, again, all failed to show a convincing co-localization of the three channels at the same depth. These results argue against a CF-GoC connection in the GL.

### Quantification in thick sections

**Figure 4:**
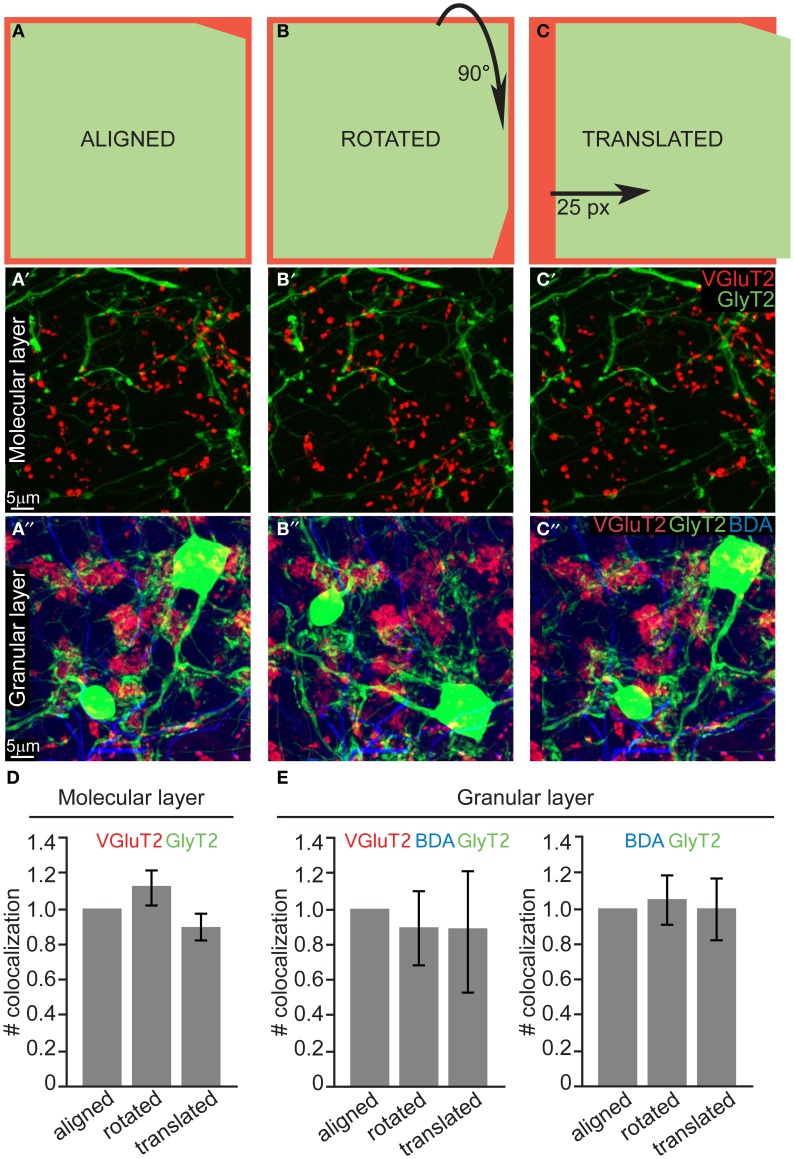
**Quantification of potential colocalization in thick slices also returns a negative result. (A)** Schematic drawing of the “aligned” configuration, and examples of maximum intensity projections of 10μ m slices from both the ML and GL (**A'** and **A”**, respectively). **(B–B”)** Same as **(A–A”)** for the “rotated” configuration, in which one channel was rotated 90° clockwise. **(C–C”)** Same as **(A–A”)** for the “translated” configuration in which one channel was shifted 25 pixels to the right. **(D)** Quantification of the number of colocalization between VGluT2 and mGluR2/GlyT2 found in the ML (*n* = 30, *N* = 3). **(E)** Quantification of the number of colocalization between VGluT2, BDA, and GlyT2 or between BDA and GlyT2 only found in the GL (*n* = 30, *N* = 3). The values are presented in (**D** and **E**) as mean ± s.e.m. and are normalized to the respective aligned configurations for averaging purposes.

Our systematic analysis in the z-plane (optical sections of 0.3–0.4 μm) returned negative results both for both layers. As a final test to confirm that due to the above mentioned thinness of slices we did not miss any contact (i.e., fibers lying just above or just below the cell body or dendrite), we performed an additional analysis on maximum projection of thick slices (10 μm). The bidimensional flattening of such volume is due to produce noise, which can be used as a statistical comparison to test for signal (i.e., real connections). Specifically, to confirm the absence of CF-GoC contacts and prove that all the colocalization that we saw in such maximum projections were indeed noise, we rotated and translated one channel while keeping the others fixed and blindly counted the colocalizations in all configurations (aligned, rotated, and translated; see Figures 4A–C and Methods). In the ML the number of colocalizations between VGluT2 and GlyT2/mGluR2 identified in the aligned configuration did not differ from the ones identified in the rotated nor in the translated ones (Figure 4D; aligned vs. rotated *p* = 0.46, aligned vs. translated *p* = 0.62; One-Way ANOVA with Tukey correction; data normalized to the aligned configuration). In the GL we counted both the triple colocalization VGluT2 + Gly + BDA (which were very scarce) and the double GlyT2 + BDA, and again we found no difference between the three configurations (Figure 4E; all *p* > 0.93 One-Way ANOVA with Tukey correction; data normalized to the aligned configuration). This lack of difference suggests that indeed in tick sections the colocalization are noise, and complements our thin-sections analysis in indicating that there are no connections between CF and GoC.

## Discussion

The present study addresses with morphological techniques the long-standing question as to whether olivary CFs contact cerebellar GoCs. Our analyses failed to prove such an existence in both the molecular layer and granular layer, whereas we found robust evidence for appositions of CFs at PCs in the molecular layer. While we clearly and consistently encountered co-localizations of GoC soma or dendrites with anterogradely traced CFs, these apposition points never also stained positively for the synaptic marker VGluT2 (again, unlike the VGluT2 labeling in the molecular layer onto PCs' dendrites and MLIs' cell-bodies). This observation could potentially explain some of the earlier reports supporting the connection, in which standard light microscopy (LM) techniques were employed to trace CFs without co-staining for synaptic proteins such as VGluT2 (Scheibel and Scheibel, 1954; Sugihara et al., 1999; Shinoda et al., 2000).

Three different laboratories have presented electron microscopic (EM) analysis corroborating the existence of CF-GoC contacts (Hamori and Szentagothai, 1966b; Desclin, 1976; Castejon and Castejon, 2000). While EM is a superior analytical technique compared to the CM that we employed, all three EM studies share the same limitation: neither GoCs nor CFs were marked with immunocytochemical labeling, leaving ample room for interpretation of the micrographs. The inconclusiveness of these studies and the clear necessity of having unequivocally marked presynaptic and postsynaptic elements prompted us to apply CM, and with these stringent conditions we consistently failed to see synaptic connections. While being aware that it is hardly possible to demonstrate the non-existence of any entity, our data strongly suggest the absence of direct CF-GoC contacts.

Our findings reopen the debate about both the nature of the Scheibel collaterals, and, more importantly, the interpretation of the *in vivo* electrophysiological experiments performed by Schulman and Bloom (1981) in the Eighties and recently confirmed by Xu and Edgley (2008). Regarding the first issue, while their existence is undoubted, it is unclear what function such projections might serve. Either they contact cells other than GoCs, or, they might be vestigial “loser” CFs that retracted their synapses from PCs soma during the developmental competition and were not fully degraded to the branching point (Sugihara, 2006). With respect to the *in vivo* results, both studies mentioned above demonstrated that olivary stimulation decreases the firing rate of GoCs, an effect that if not due to a direct contact mediated by the abundantly-expressed mGluR2 receptors (Watanabe and Nakanishi, 2003), has to be derived either from extrasynaptic signaling or from an inhibitory intermediary neuron receiving CF input and projecting onto a GoC (Xu and Edgley, 2008). Which neurons are the candidates for this intermediary role? It is now widely acknowledged that both MLIs and PCs receive functional CF inputs (Ramon y Cajal, 1995; Schmolesky et al., 2002; Szapiro and Barbour, 2007; Mathews et al., 2012). Until recently the MLI would have been considered the best candidate to fulfill the presumptive inhibitory, intermediary role, because it was traditionally thought to project to GoCs (Ramon y Cajal, 1995; Dumoulin et al., 2001). However, a recent physiological study failed to prove a functional connection between MLIs and GoCs (Hull and Regehr, 2012). Maybe, PCs form a better candidate. According to an ultrastructural study by Hamori and Szentagothai (1966a) and a CM study by Frola and colleagues (Frola et al., 2012) axon collaterals of PCs do contact GoC cell-bodies. If this preliminary evidence can be confirmed with paired recordings, they could explain the electrophysiological results *in vivo* and open new scenarios in terms of cerebellar computation and integration at the input stage (D'Angelo and De Zeeuw, 2009).

### Conflict of interest statement

The authors declare that the research was conducted in the absence of any commercial or financial relationships that could be construed as a potential conflict of interest.

## References

[B1] Albus J. S. (1971). A theory of cerebellar function. Math. Biosci. 10, 25–61

[B2] Bloedel J. R. Bracha V. (1998). Current concepts of climbing fiber function. Anat. Rec. 253, 118–126 10.1002/(SICI)1097-0185(199808)253:4<118::AID-AR7>3.0.CO;2-P 9740035

[B3] Castejon O. J. Castejon H. V. (2000). Correlative microscopy of cerebellar Golgi cells. Biocell 24, 13–30 10893796

[B4] D'Angelo E. De Zeeuw C. I. (2009). Timing and plasticity in the cerebellum: focus on the granular layer. Trends Neurosci. 32, 30–40 10.1016/j.tins.2008.09.007 18977038

[B5] Desclin J. C. (1976). Early terminal degeneration of cerebellar climbing fibers after destruction of the inferior olive in the rat. Synaptic relationships in the molecular layer. Anat. Embryol. (Berl.) 149, 87–112 1267191 10.1007/BF00315087

[B6] De Zeeuw C. I. Hoebeek F. E. Bosman L. W. Schonewille M. Witter L. Koekkoek S. K. (2011). Spatiotemporal firing patterns in the cerebellum. Nat. Rev. Neurosci. 12, 327–344 10.1038/nrn3011 21544091

[B7] Dumoulin A. Triller A. Dieudonne S. (2001). IPSC kinetics at identified GABAergic and mixed GABAergic and glycinergic synapses onto cerebellar Golgi cells. J. Neurosci. 21, 6045–6057 11487628 10.1523/JNEUROSCI.21-16-06045.2001 PMC6763194

[B8] Frola E. Patrizi A. Sassoè-Pognetto M. (2012). Novel Gabaergic Inputs to Golgi Cells in the Cerebellar Granule Cell Layer, 8th FENS 3782. (Barcelona), 119.08.

[B9] Galliano E. Mazzarello P. D'Angelo E. (2010). Discovery and rediscoveries of Golgi cells. J. Physiol. 588, 3639–3655 10.1113/jphysiol.2010.189605 20581044 PMC2998217

[B10] Gao Z. van Beugen B. J. De Zeeuw C. I. (2012). Distributed synergistic plasticity and cerebellar learning. Nat. Rev. Neurosci. 13, 619–635 10.1038/nrn3312 22895474

[B11] Hamori J. Szentagothai J. (1966a). Identification under the electron microscope of climbing fibers and their synaptic contacts. Exp. Brain Res. 1, 65–81 5910944 10.1007/BF00235210

[B12] Hamori J. Szentagothai J. (1966b). Participation of Golgi neuron processes in the cerebellar glomeruli: an electron microscope study. Exp. Brain Res. 2, 35–48 5921132 10.1007/BF00234359

[B13] Hossaini M. Cardona Cano S. van Dis V. Haasdijk E. D. Hoogenraad C. C. Holstege J. C. (2011). Spinal inhibitory interneuron pathology follows motor neuron degeneration independent of glial mutant superoxide dismutase 1 expression in SOD1-ALS mice. J. Neuropathol. Exp. Neurol. 70, 662–677 10.1097/NEN.0b013e31822581ac 21760539

[B14] Hull C. Regehr W. G. (2012). Identification of an inhibitory circuit that regulates cerebellar Golgi cell activity. Neuron 73, 149–158 10.1016/j.neuron.2011.10.030 22243753 PMC3259536

[B15] Kaneko T. Fujiyama F. Hioki H. (2002). Immunohistochemical localization of candidates for vesicular glutamate transporters in the rat brain. J. Comp. Neurol. 444, 39–62 10.1002/cne.10129 11835181

[B16] Marr D. (1969). A theory of cerebellar cortex. J. Physiol. 202, 437–470 5784296 10.1113/jphysiol.1969.sp008820 PMC1351491

[B17] Mathews P. J. Lee K. H. Peng Z. Houser C. R. Otis T. S. (2012). Effects of climbing fiber driven inhibition on purkinje neuron spiking. J. Neurosci. 32, 17988–17997 10.1523/JNEUROSCI.3916-12.2012 23238715 PMC3532857

[B18] Neki A. Ohishi H. Kaneko T. Shigemoto R. Nakanishi S. Mizuno N. (1996). Metabotropic glutamate receptors mGluR2 and mGluR5 are expressed in two non-overlapping populations of Golgi cells in the rat cerebellum. Neuroscience 75, 815–826 10.1016/0306-4522(96)00316-8 8951875

[B19] Palay S. L. Chan-Palay V. (1974). Cerebellar Cortex: Cytology and Organization. Berlin; Heidelberg; New York: Springer

[B20] Paxinos G. Franklin K. B. J. (2004). The Mouse Brain in Stereotaxic Coordinates. San Diego, CA: Academic Press

[B21] Pijpers A. Voogd J. Ruigrok T. J. (2005). Topography of olivo-cortico-nuclear modules in the intermediate cerebellum of the rat. J. Comp. Neurol. 492, 193–213 10.1002/cne.20707 16196034

[B22] Ramon y Cajal S. (1995). Histology of the Nervous System of Man and Vertebrates. New York, NY: Oxford University Press

[B23] Ruigrok T. J. Teune T. M. van der Burg J. Sabel-Goedknegt H. (1995). A retrograde double-labeling technique for light microscopy. A combination of axonal transport of cholera toxin B-subunit and a gold-lectin conjugate. J. Neurosci. Methods 61, 127–138 10.1016/0165-0270(94)00034-E 8618410

[B24] Scheibel M. E. Scheibel A. B. (1954). Observations on the intracortical relations of the climbing fibers of the cerebellum; a Golgi study. J. Comp. Neurol. 101, 733–763 13233358 10.1002/cne.901010305

[B25] Schmolesky M. T. Weber J. T. De Zeeuw C. I. Hansel C. (2002). The making of a complex spike: ionic composition and plasticity. Ann. N.Y. Acad. Sci. 978, 359–390 10.1111/j.1749-6632.2002.tb07581.x 12582067

[B26] Schulman J. A. Bloom F. E. (1981). Golgi cells of the cerebellum are inhibited by inferior olive activity. Brain Res. 210, 350–355 10.1016/0006-8993(81)90908-2 7225813

[B27] Shinoda Y. Sugihara I. Wu H. S. Sugiuchi Y. (2000). The entire trajectory of single climbing and mossy fibers in the cerebellar nuclei and cortex. Prog. Brain Res. 124, 173–186 10.1016/S0079-6123(00)24015-6 10943124

[B28] Simat M. Parpan F. Fritschy J. M. (2007). Heterogeneity of glycinergic and gabaergic interneurons in the granule cell layer of mouse cerebellum. J. Comp. Neurol. 500, 71–83 10.1002/cne.21142 17099896

[B29] Sugihara I. (2006). Organization and remodeling of the olivocerebellar climbing fiber projection. Cerebellum 5, 15–22 10.1080/14734220500527385 16527759

[B30] Sugihara I. Wu H. Shinoda Y. (1999). Morphology of single olivocerebellar axons labeled with biotinylated dextran amine in the rat. J. Comp. Neurol. 414, 131–148 10.1002/(SICI)1096-9861(19991115)414:2<131::AID-CNE1>3.0.CO;2-F 10516588

[B31] Szapiro G. Barbour B. (2007). Multiple climbing fibers signal to molecular layer interneurons exclusively via glutamate spillover. Nat. Neurosci. 10, 735–742 10.1038/nn1907 17515900

[B32] Watanabe D. Nakanishi S. (2003). mGluR2 postsynaptically senses granule cell inputs at Golgi cell synapses. Neuron 39, 821–829 10.1016/S0896-6273(03)00530-0 12948448

[B33] Xu W. Edgley S. A. (2008). Climbing fibre-dependent changes in Golgi cell responses to peripheral stimulation. J. Physiol. 586, 4951–4959 10.1113/jphysiol.2008.160879 18755742 PMC2614060

[B34] Zeilhofer H. U. Studler B. Arabadzisz D. Schweizer C. Ahmadi S. Layh B. (2005). Glycinergic neurons expressing enhanced green fluorescent protein in bacterial artificial chromosome transgenic mice. J. Comp. Neurol. 482, 123–141 10.1002/cne.20349 15611994

